# Entropy-Based Emotion Recognition from Multichannel EEG Signals Using Artificial Neural Network

**DOI:** 10.1155/2022/6000989

**Published:** 2022-10-13

**Authors:** Si Thu Aung, Mehedi Hassan, Mark Brady, Zubaer Ibna Mannan, Sami Azam, Asif Karim, Sadika Zaman, Yodchanan Wongsawat

**Affiliations:** ^1^Department of Biomedical Engineering, Faculty of Engineering, Mahidol University, Salaya, Thailand; ^2^Computer Science and Engineering, North Western University, Khulna, Bangladesh; ^3^Asia Pacific College of Business and Law, Charles Darwin University, Casuarina, NT, Australia; ^4^Department of Smart Computing, Kyungdong University, Global Campus, Goseong-Gun, Republic of Korea; ^5^College of Engineering IT and Environment, Charles Darwin University, Casuarina, NT, Australia

## Abstract

Humans experience a variety of emotions throughout the course of their daily lives, including happiness, sadness, and rage. As a result, an effective emotion identification system is essential for electroencephalography (EEG) data to accurately reflect emotion in real-time. Although recent studies on this problem can provide acceptable performance measures, it is still not adequate for the implementation of a complete emotion recognition system. In this research work, we propose a new approach for an emotion recognition system, using multichannel EEG calculation with our developed entropy known as *multivariate multiscale modified-distribution entropy* (*MM-mDistEn*) which is combined with a model based on an artificial neural network (ANN) to attain a better outcome over existing methods. The proposed system has been tested with two different datasets and achieved better accuracy than existing methods. For the GAMEEMO dataset, we achieved an average accuracy ± standard deviation of 95.73% ± 0.67 for valence and 96.78% ± 0.25 for arousal. Moreover, the average accuracy percentage for the DEAP dataset reached 92.57% ± 1.51 in valence and 80.23% ± 1.83 in arousal.

## 1. Introduction

Emotions play an important role in our day-to-day activities, including communication, decision-making, and personal development [1]. Moreover, an emotion recognition system is not only important for healthy people but also for disabled people to detect emotional changes and is used for a variety of applications. Therefore, the system requires a better performance measure to accurately detect emotional changes in humans. The human emotion recognition system is part of the artificial intelligence (AI) field [2, 3], and this system includes the procedures of data processing, interpreting, and identifying emotional states [4]. The continuous development of AI technology, including deep learning and machine learning, is combined with an advanced clinical treatment which has helped to improve the classification of human emotion in recent years [5].

Human emotion can be recognized in different ways, such as facial expression [6, 7], speech [8], and physiological signals, which are some of the better ways to recognize human emotion [9]. Researchers and scientists are becoming more and more interested in implementing an emotion recognition system using EEG signals [10, 11]. This is because emotion recognition systems have applications in several areas including brain-computer interface (BCI), healthcare, and E-learning systems [12]. In terms of BCI and healthcare, the emotion recognition system plays a main role in helping disabled patients, who cannot communicate directly with healthcare providers, where they can use their emotions for communication [13]. Moreover, the real-time advantage of EEG signal is in helping to detect the emotions of humans and their mental states [1]. Therefore, the emotion recognition system is important, not only for healthy people, to detect the changes in emotion in real-time, but also for disabled people, in helping improve the communication between patients and healthcare providers.

There are several significant problems found with current emotion recognition systems. The main concern of the recognition system is that it should provide a better classification performance measure in terms of accuracy percentage, to classify changes in human emotion from time to time. Most emotion recognition systems use EEG signals to recognize human emotion. Hence, the traditional EEG signal processing system uses time-domain [14], frequency-domain [15], and time-frequency analysis [16] as feature extraction methods to obtain important information from the EEG signals. These feature extraction methods can achieve good classification accuracy but are yet to achieve a better emotion recognition system. Therefore, we propose a new emotion recognition system as follows:

- A new entropy method called multivariate multiscale modified-distribution entropy (MM-mDistEn) has been developed.
- The proposed system has been combined with an artificial neural network (ANN) to achieve better performance measures over existing methods.

## 2. Related Works

In Reference [15], a deep learning network (DLN) is constructed with a Stacked Autoencoder (SAE) with hierarchical feature learning approach to classify the different levels of arousal and valence. The study demonstrated an accuracy of 46.03% for arousal and 49.52% for valence [17]. However, the principal component analysis (PCA) has been put into use to extract important features and minimize the nonstationary effect of the EEG signal, and then the accuracies of valence and arousal are improved to 5.55% and 6.53%, respectively. Reference [18] discussed a binary classification technique for emotion detection that utilizes sample entropy and empirical mode decomposition (EMD), and the work reported an accuracy of 94.98%.

In Reference [19], the EEG signal characteristics have been extracted using the power spectral density (PSD), and human emotions are identified using the deep neural network (DNN). The accuracy of this study has been shown to be 82.0% for both classes of valence and arousal [19]. To classify emotions based on their valence and arousal, machine learning models [20] such as bagging trees (BT), support vector machines (SVM), linear discriminant analysis (LDA), Bayesian linear discriminant analysis (BLDA) models, and deep convolutional neural networks (CNN) are used. Deep CNN achieved the best recognition performance on features that combined temporal and frequency information [21]. The DEAP [22] dataset has been used in all these research studies.

## 3. Methodology

In our emotion recognition system, multivariate entropy is used for the extraction of important features from the multichannel EEG signal. Recording the EEG signal from the human brain using one or two channels is not enough to provide sufficient information about human emotion, and therefore, the multivariate approach is an alternate research approach for the analysis of multichannel EEG signals. In this research, MM-mDistEn is used as a feature extraction method. This method achieved good performance measures to provide not only for classification but also for the prediction of epileptic EEG signals [23]. Therefore, MM-mDistEn is employed in this method to take advantage of multivariate entropy calculation, and the calculated entropy values have been used for the next step of emotion classification. The classification between the valence and arousal of the emotion EEG signal is achieved using ANN. There are three steps to calculate the MM-mDistEn, and these represent the construction of multivariate time series, a coarse-graining process.

### 3.1. Multivariate Multiscale Modified-Distribution Entropy (MM-mDistEn)

The algorithm is constructed as follows:

Step 1. Multivariate time series:
Firstly, we developed the multivariate time series from the given time series data. The calculation is shown in (1):
  (1) $$\begin{array}{c} X={\left\{x_{c,i}\right\}}_{i=1}^{N}\text{,} \end{array}$$
where *c* is the number of channels (variables) and *N* is the number of samples in each channel.
Step 2. Coarse-graining process:
According to the scale factor, the multivariate time series data may be used to generate the coarse-grained time series, and the equation can be expressed as follows:
  (2) $$\begin{array}{c} g_{c,j}^{N_{s}}=\frac{1}{s}\overset{j∙s}{\underset{i=\left(j-1\right)s+1}{\sum }}x_{c,j},\left(1\leq j\leq N_{s}\right)\text{,} \end{array}$$
where *g*_*c*,*j*_^*N*_*s*_^ is the multivariate coarse-grained time series, *s* is a scale factor, *c* is the number of channels (variables), and *N* is the number of samples (*N*_*s*_=(*N*/*s*)).
Step 3. Calculate multivariate multiscale modified-distribution entropy:
Before determining the entropy values, we perform the phase-space reconstruction, and the reconstruction is as follows:
  (3) $$\begin{array}{c} M_{m}^{s}\left(j\right)=\left[\begin{array}{cccc} g_{1,j}^{s} & g_{1,j+\tau_{1}}^{s} & \ldots & g_{1,j+\left(m_{1}-1\right)\tau_{1}}^{s}\\ g_{2,j}^{s} & g_{2,j+\tau_{2}}^{s} & \ldots & g_{2,j+\left(m_{2}-1\right)\tau_{2}}^{s}\\ \vdots & \vdots & \vdots & \vdots \\ g_{c,j}^{s} & g_{1,j+\tau_{c}}^{s} & \ldots & g_{c,j+\left(m_{c}-1\right)\tau_{c}}^{s} \end{array}\right]\text{,}\left(1\leq j\leq {\;N}_{s}\right)\text{,} \end{array}$$
where *m* and *τ* are the embedding dimension and time delay, respectively. For this research, we used the *m* = 3 and *τ* = 1 (see more information in parameter selection). In our newly formulated approach, MM-mDistEn, which is based on distribution entropy, two additional threshold parameters, “*r*” and “*n*,” are added to the existing parameters. The number of *n* is set to 2, whereas the standard deviation of all the data values is multiplied by 0.2 to determine the value of *r* [24].

MM-mDistEn can be calculated by using the following equation:

(4) $$\begin{array}{c} MM-mDistEn\left(m,\tau ,r,n,B,s\right)=-\frac{1}{\log_{2}\left(B\right)}\overset{B}{\underset{t=1}{\sum }}P_{t}\left(D_{ij}^{s}\right)\log_{2}\;\left[P_{t}\left(D_{ij}^{s}\right)\right],1\leq i,j\leq m--\;1,i\neq j\text{.} \end{array}$$

**Figure 1:**
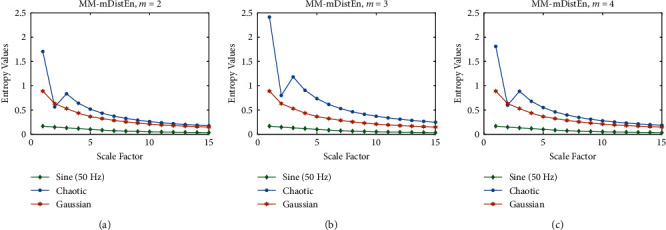
Entropy values (no unit) of simulation data as a function of different scale factors using three embedding dimensions: (a) *m* = 2, (b) *m* = 3, and (c) *m* = 4.

**Figure 2:**
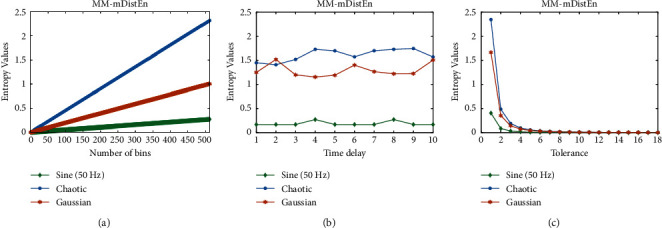
Entropy values (no unit) of simulation data: (a) number of bins (*B*) (20 to 29), (b) time delay (*τ*) (1 to 10), and (c) tolerance (*r*).

Predefined values are used for the selection parameters in MM-mDistEn. To calculate the entropy values, a total of five parameter values are needed. The proposed entropy method's optimal parameter values are determined using simulation data, and three separate series, the chaotic series, and the Gaussian series are each employed [23]. There are 400 samples in each series. First, we reconstruct the phase-space using the time delay (*τ*) and dimension (*m*), whose values are 1 and 3, respectively [25]. Because this value can distinguish between the three data series, the embedding dimension value in Figure 1 has been set to 3. The distance matrix (**D**_*ij*_) is then constructed with the parameters *r* and *n*, where *r* is the standard deviation of the series multiplied by 0.2 and *n* is set to 2 [26]. The parameter *r* is the time series' standard deviation multiplied by 2, and *n* is equal to 2, because in Figure 2 a big *r* and *n* value can affect noise while a small *r* and *n* value can result in information loss [24]. A further parameter value known as the bin number (*B*) is required when calculating the empirical probability density function (ePDF), and B is set to 64 for our estimation [27]. The dimension (*m*), which we left at the same value as before, and two extra parameters, the breadth of the fuzzy exponential function and the step of the fuzzy exponential function, which we left at 0.3 and 2, respectively, are required for the computation of fuzzy entropy [26]. The scale factor (*s*) is also required because we calculated the multivariate multiscale entropy values, and the scale values utilised in our investigation ranged from 1 to 15 [28].

**Figure 3:**
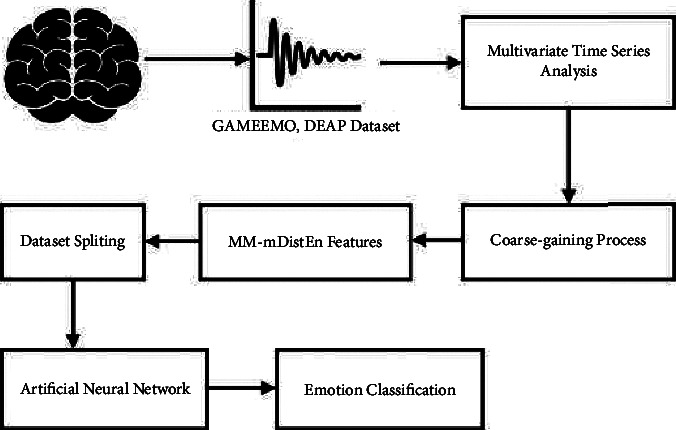
Flow diagram of emotion classification using multivariate EEG signals.

**Figure 4:**
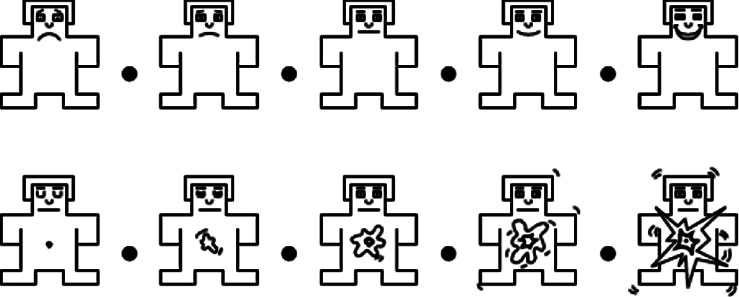
From the top to bottom, human emotion states are valence and arousal [30].

The emotion classification system has been implemented using MM-mDistEn for feature extraction, and ANN is used for the classification of two classes: valence and arousal. The flow diagram is shown in Figure 3. First, the EEG raw data is reconstructed into multivariate time series and the coarse-graining process is also applied to get the multiscale time series. After getting MM-mDistEn features, these features are split into the training dataset and testing dataset. Backpropagation is used to train the ANN model, and the RMSprop algorithm is used for optimisation [29]. The rectified linear unit (ReLU) activation function [30] has been deployed for the hidden layers to introduce nonlinearity and improve robustness. The loss function in this model is the binary crossentropy used to evaluate the binary classification problem. The performance of the ANN model has then been estimated using the cross-validation procedure utilising 10-fold cross-validation [28, 31]. For both datasets, the networks for each person have been trained separately. The level of arousal/valence is categorised as high if the score for each topic is more than 4.5. The level of arousal/valence is categorised as low [32] if each subject receives a score of less than 4.5.  Figure4 showed the accuracy performance measure of our system.  Table 1 described the comparison between our system and some previous research works.

In this research, two different emotion EEG datasets are used to show the performance of the proposed recognition system. They are a database for emotion recognition systems based on EEG signals and various computer games (GAMEEMO) and a database for emotion analysis using physiological signals (DEAP). A detailed description will be provided for each dataset in the following section.

### 3.2. Database for Emotion Recognition System Based on EEG Signals and Various Computer Games (GAMEEMO)

**Figure 5:**
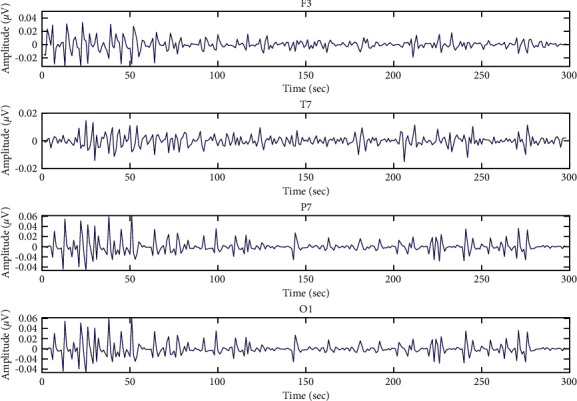
Emotion EEG signals from subject No. 1.

The first dataset, GAMEEMO, was composed of 28 subjects, with ages ranging from 20 to 27 years with good health conditions and no disease history [33]. Each subject played four computer games for 5 min to measure funny, boring, horror, and calm emotions. In this dataset, they used a 14-channel EEG device and established a connection using a Wi-Fi network. The sampling rate is 128 Hz, and the bandwidth of the EEG signal is between 0.16 Hz and 43 Hz and included two types of datasets: raw and preprocessed data. For preprocessed data, they used the fifth-order sinc filter to remove artifacts resulting from the movement of hands, head, and arms. In this research proposal, the preprocessed data is used for the analysis of emotion EEG signal and the visualization of the emotion EEG signal from subject No. 1 with the different areas of the human brain, including frontal, temporal, parietal, and occipital, which is shown in Figure 5.

### 3.3. Database for Emotion Analysis using Physiological Signals (DEAP)

32 healthy subjects with an average age of 26.9 years are recorded for 32-channel EEG and 8-channel peripheral physiological signals in the DEAP database [22]. Each participant had to watch 40 one-minute-long music video snippets and rate them based on their valence, arousal, dominance, likeability, and familiarity. The sampling rate of this dataset is 128 Hz, and the signal is applied with the band-pass filter of the frequency of 4.0 to 45 Hz. Independent component analysis (ICA) is applied to eliminate EOG noise in the DEAP dataset to ensure the data can accurately represent the emotion of the participants.

**Figure 6:**
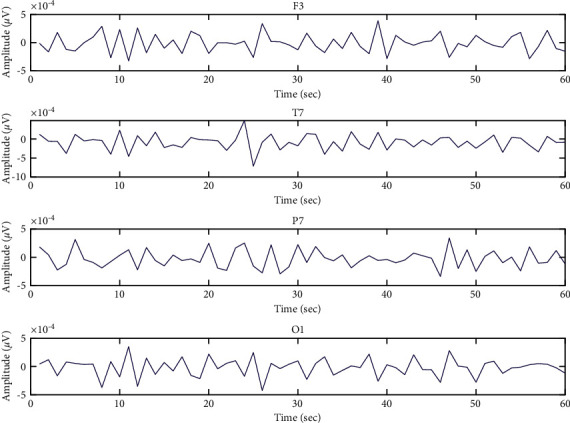
Emotion EEG signals from the first trail of subject No. 1.

Figure 4 shows the human emotional states which augment personal ratings from left to right [30]. In this research work, the 3 s pretrials have been removed from the 63 s trials, and the 60 s trials have been used for the analysis of the emotion EEG signal. The first trail of subject No. 1 with the different areas of the human brain, including frontal, temporal, parietal, and occipital, is visualized in Figure 6.

## 4. Results and Discussion

**Figure 7:**
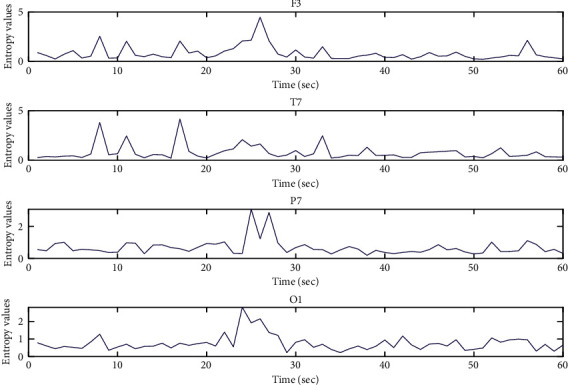
MM-mDistEn values for emotion EEG signals from the DEAP dataset (subject no. 1).

**Figure 8:**
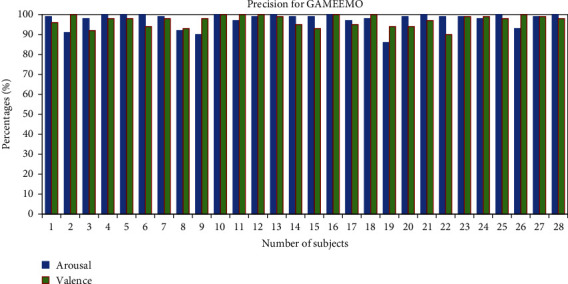
Precision percentage of all subjects for the GAMEEMO dataset.

**Figure 9:**
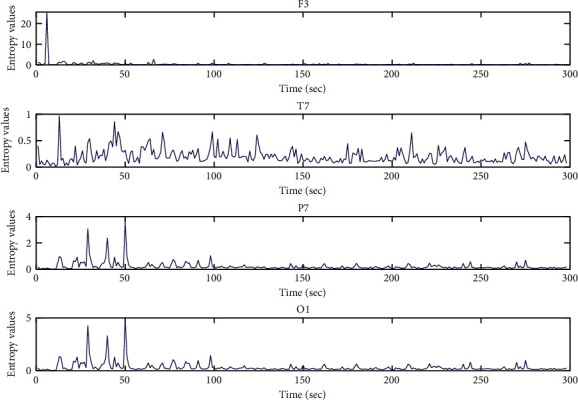
MM-mDistEn values for emotion EEG signals from the GAMEEMO dataset (subject no. 1).

Both Figures 7 and 8 are the data visualization of the entropy values of subject No. 1 from GAMEEMO and DEAP. These figures illustrate the patterns of what an emotional EEG signal looks like in entropy values in the different areas of the human brain, including frontal, temporal, parietal, and occipital. It can be clearly seen that the difference between the original EEG signal (see Figures 5 and 6) and the calculated entropy values of the emotion signals (see Figures 7 and 9) from the different areas of the brain. The peak calculated entropy values indicate a high intensity of human emotion in those periodsdue to the nature of the entropy which can reveal the high entropy values for irregularity in the time-series signal [26].

**Figure 10:**
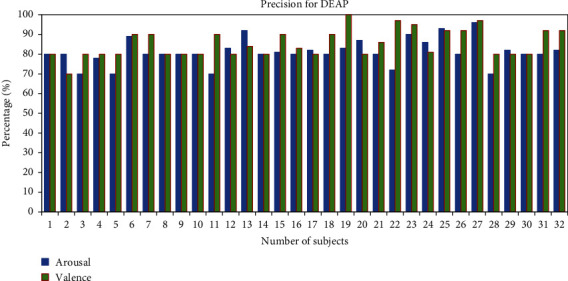
Precision percentage of all subjects for the DEAP dataset.

Performance is evaluated based on two datasets by calculating precision, recall, *F*1-score, and accuracy:

(5) $$\begin{array}{c} Precision\left(\%\right)=\frac{TP}{TP+FP}\times 100\text{,}\\ Recall\left(\%\right)=\frac{TP}{TP+FN}\times 100\text{,}\\ F1\;score\left(\%\right)=\frac{2*TP}{2*TP+FP+FN}\times 100\text{,}\\ Accuracy\left(\%\right)=\frac{TP+TN}{TP+FP+TN+FN}\times 100\text{,} \end{array}$$

where TN is the number of true negatives, TP is the number of true positives, and FN and FP are the number of false negatives [31] and false positives, respectively [31]. We calculated the precision of individual subjects for the classification of human emotions. It clearly shows that the average precision percentage of the classification of two classes for all the 28 subjects from the GAMEEMO dataset has been found to be 97.03% for valence and 98% for arousal as shown in Figure 8. Moreover, the average precision percentage from the DEAP dataset for all 32 subjects has been 85.34% in valence and 81.12% in arousal as shown in Figure 10.

**Figure 11:**
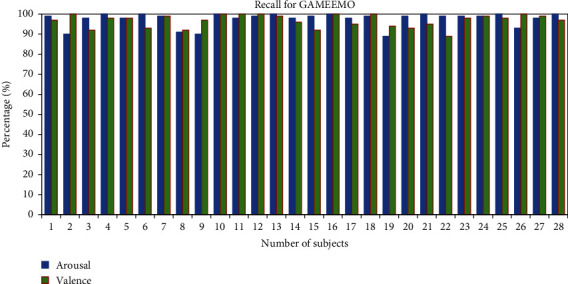
Recall percentage of all subjects for the GAMEEMO dataset.

**Figure 12:**
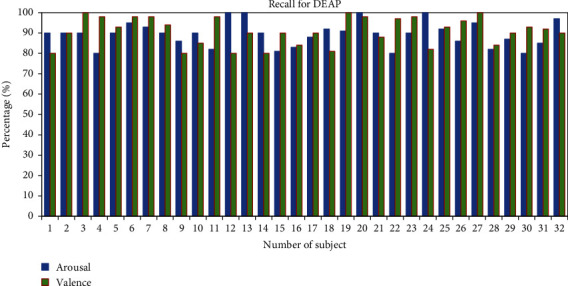
Recall percentage of all subjects for the DEAP dataset.

The recall of individual subjects has been calculated for the classification of human emotions. The average recall percentage of the two classes for all the 28 subjects is found to be 95.78% for valence and 97.57% for arousal, as shown in Figure 11. In addition, the average recall percentage from the DEAP dataset for all 32 subjects is 90.93% in valence and 89.53% in arousal as shown in Figure 12.

**Figure 13:**
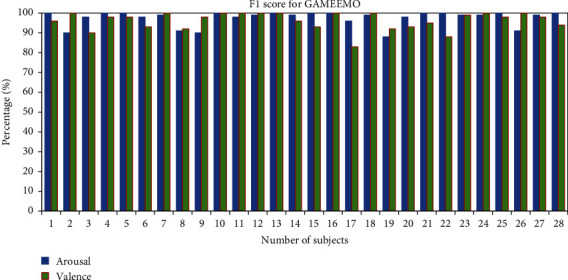
*F*1-score percentage of all subjects for the GAMEEMO dataset.

**Figure 14:**
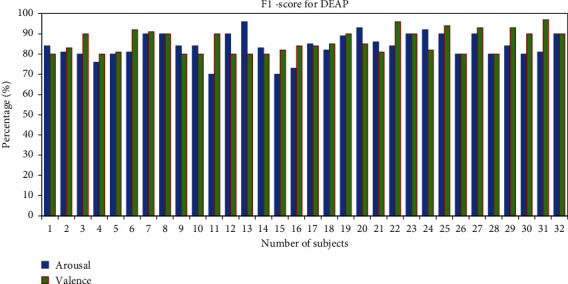
*F*1-score percentage of all subjects for the DEAP dataset.

The overall *F*1-score of the subjects has been calculated to measure the classification of emotions. The average *F*1-score for classifications of two classes for all 28 subjects from the GAMEEMO dataset is 96.21% for valence and 97.53% for arousal (see Figure 13). Furthermore, the average percentage of *F*1-scores from the DEAP dataset for all 32 subjects is 86.03% in valence and 84% in arousal as shown in Figure 14.

**Figure 15:**
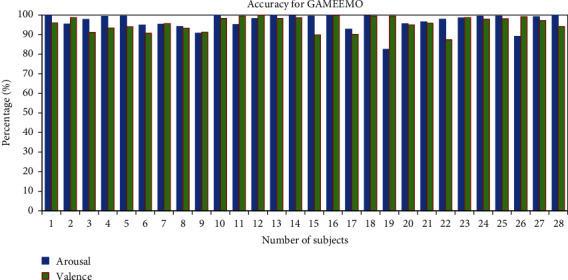
Accuracy percentage of all subjects for the GAMEEMO dataset.

**Figure 16:**
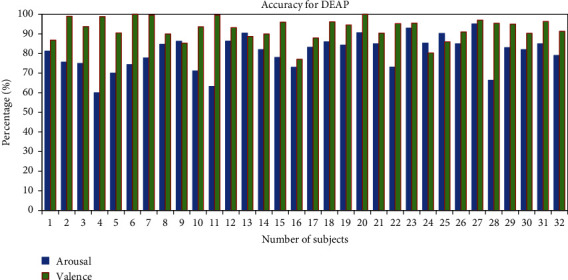
Accuracy percentage of all subjects for the DEAP dataset.

To find out how accurately human emotions are classified, we calculated the accuracy of individual subjects. The average accuracy percentage for valence and arousal of the two classes has been 95.79% for all 28 subjects from the GAMEEMO dataset as shown in Figure 15. Moreover, the average accuracy percentage of the DEAP dataset for all 32 subjects has been 90.26% in valence and 80.48% in arousal as shown in Figure 16.

**Figure 17:**
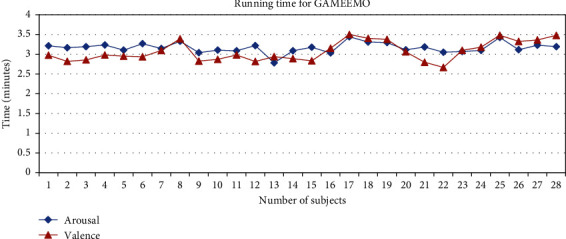
Running time of all subjects for the GAMEEMO dataset.

**Figure 18:**
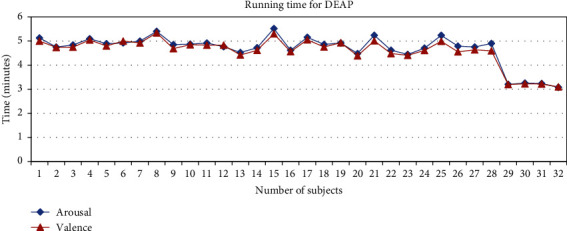
Running time of all subjects for the DEAP dataset.

The training time for each dataset is illustrated in Figures 17 and 18. For the GAMEEMO dataset, the average running time for valence classification is 3.16 minutes while arousal classification is 3.07 minutes. On the other hand, the average running time for valence and arousal from the DEAP dataset are 4.58 and 4.67 minutes, respectively. Data analysis is done offline with Python and MATLAB (R2019a, The MathWorks, Natwick, MA) (3.9.7).

In Table 1, it is clearly shown that our proposed method achieves better accuracy measures than existing methods such as discrete wavelet transform (DWT) with multilayer perceptron neural network (MLPNN) and spectral entropy calculation with a deep learning model of bidirectional long-short term memory (BiLSTM) [29, 31]. The average accuracy of our proposed method is smaller than the prime pattern network with a support vector machine (SVM) [34], but our proposed system used the multichannel approach to calculate the features from all 14 channels of EEG.

The comparison of our proposed emotion recognition system and others studies on the same dataset of DEAP is shown in Table 2. These recent studies include the frequency band power with LSTM-RNN, frequency band with CNN and time, wavelet, and frequency with SVM. Our proposed emotion recognition system achieved the highest accuracy percentage in valence and arousal. Although the accuracy of our model for arousal is less than that of CNN model because we used fewer parameters for the implementation of CNN than our ANN model [22] and all accuracy percentages are shown in Table 2.

## 5. Conclusion

In this research, we proposed an alternative approach to an emotion recognition system using our developed method called *MM-mDistEn* which is combined with the powerful classification algorithm as an ANN. We proved that our system achieved better accuracy performance not only for the GAMEEMO dataset but also for the DEAP dataset. Therefore, our proposed system significantly improves the performance of emotion recognition compared with other existing methods. For further studies, we still need to analyze the several emotion classes instead of the two classes: valence and arousal that are used in our proposed system.

## Figures and Tables

**Table 1:** Comparison of results with other studies on the GAMEEMO dataset.

Reference	Feature extraction method	Classifier	Accuracy (%) valence	Accuracy (%) arousal
[33]	DWT	MLPNN	82.0	94.6
[32]	Spectral entropy	BiLSTM	76.93	—
[34]	Prime pattern network	SVM	100	—
Our work	Multivariate entropy	ANN	95.73	96.78

**Table 2:** Comparison of results with other studies on the DEAP dataset.

Reference	Feature extraction method	Classifier	Accuracy (%)	Accuracy (%)
			Valence	Arousal
[1]	Time, wavelet, and frequency	SVM	65.92	
[35]	Frequency band	CNN	90.26	88.9
[36]	Frequency band power	LSTM-RNN	81.10	74.38
Our work	Multivariate entropy	ANN	92.57	80.23

## Data Availability

The GAMEEMO dataset can be obtained from https://data.mendeley.com/datasets/b3pn4kwpmn. The DEAP dataset can be downloaded from: https://www.eecs.qmul.ac.uk/mmv/datasets/deap/. Both GAMEEMO and DEAP databases have been used for this study.

## References

[1] Khateeb M., Anwar S. M., Alnowami M. (2021). Multi-domain feature fusion for emotionclassification using DEAP dataset. *IEEE Access*.

[2] EL Merrassi W., Abounada A., Ramzi M. (2022). Performance analysis of novel robust ANN-MRAS observer applied to induction motor drive. *International Journal of System Assurance Engineering and Management*.

[3] El Merrassi W., Abounada A., Ramzi M. (2021). Advanced speed sensorless control strategy for induction machine based on neuro-MRAS observer. *Materials Today Proceedings*.

[4] Erol B. A., Majumdar A., Benavidez P., Rad P., Choo K.K. R., Jamshidi M. (2020). Toward artificial emotional intelligence for cooperative social human–machine interaction. *IEEE Transactions on Computational Social Systems*.

[5] Dangol R., Alsadoon A., Prasad P. W. C., Seher I., Alsadoon O. H. (2020). Speech emotion recognition UsingConvolutional neural network and long-short TermMemory. *Multimedia Tools and Applications*.

[6] Mohan K., Seal A., Krejcar O., Yazidi A. (2021). Facial expression recognition using local gravitational force descriptor-based deep convolution neural networks. *IEEE Transactions on Instrumentation and Measurement*.

[7] Mohan K., Seal A., Krejcar O., Yazidi A. (2021). Fer-net: facial expression recognition using deep neural net. *Neural Computing & Applications*.

[8] Karnati M., Seal A., Yazidi A., Krejcar O. (2021). LieNet: a deep convolution neural networks framework for detecting deception. *IEEE Transactions on Cognitive and Developmental Systems*.

[9] Cohen M. X. (2017). Where does EEG come from and what does it mean?. *Trends in Neurosciences*.

[10] Fernández-Varela I., Hernández-Pereira E., Álvarez-Estévez D., Moret-Bonillo V. (2017). Combining machine learning models for the automatic detection of EEG arousals. *Neurocomputing*.

[11] Hu B., Li X., Sun S., Ratcliffe M. (2018). Attention recognition in EEG-based affective learning research using CFS+ KNN algorithm. *IEEE/ACM Transactions on Computational Biology and Bioinformatics*.

[12] Ullah H., Uzair M., Mahmood A., Ullah M., Khan S. D., Cheikh F. A. (2019). Internal emotion classification using EEG signal with sparse discriminative ensemble. *IEEE Access*.

[13] Al-Nafjan A., Alharthi K., Kurdi H. (2020). Lightweight building of an electroencephalogram-based emotion detection system. *Brain Sciences*.

[14] Liu Y., Fu G. (2021). Emotion recognition by deeply learned multi-channel textual and EEG features. *Future Generation Computer Systems*.

[15] Li M., Xu H., Liu X., Lu S. (2018). Emotion recognition from multichannel EEG signals using K-nearest neighbor classification. *Technology and Health Care*.

[16] Murugappan M., Ramachandran N., Sazali Y. (2010). Classification of human emotion from EEG using discrete wavelet transform. *Journal of Biomedical Science and Engineering*.

[17] Jirayucharoensak S., Pan-Ngum S., Israsena P. (2014). EEG-based emotion recognition using deep learning network with principal component based covariate shift adaptation. *The Scientific World Journal*.

[18] Zhang Y., Ji X., Zhang S. (2016). An approach to EEG-based emotion recognition using combined feature extraction method. *Neuroscience Letters*.

[19] Al-Nafjan A., Hosny M., Al-Wabil A., Al-Ohali Y. (2017). Classification of human emotions from electroencephalogram (EEG) signal using deep neural network. *International Journal of Advanced Computer Science and Applications*.

[20] Chowdhury A. I., Shahriar M. M., Islam A., Ahmed E., Karim A., Islam M. R. An automated system in ATM booth using face encoding and emotion recognition process.

[21] Chen J. X., Zhang P. W., Mao Z. J., Huang Y. F., Jiang D. M., Zhang Y. N. (2019). Accurate EEG-based emotion recognition on combined features using deep convolutional neural networks. *IEEE Access*.

[22] Koelstra S., Muhl C., Soleymani M. (2012). Deap: a database for emotion analysis; using physiological signals. *IEEE transactions on affective computing*.

[23] Aung S. T., Wongsawat Y. (2021). Prediction of epileptic seizures based on multivariate multiscale modified-distribution entropy. *PeerJ Computer Science*.

[24] Aung S. T., Wongsawat Y. (2020). Modified-distribution entropy as the features for the detection of epileptic seizures. *Frontiers in Physiology*.

[25] Li P., Yan C., Karmakar C., Liu C. Distribution entropy analysis of epileptic EEG signals.

[26] Chen W., Wang Z., Xie H., Yu W. (2007). Characterization of surface EMG signal based on fuzzy entropy. *IEEE Transactions on Neural Systems and Rehabilitation Engineering*.

[27] Li P., Karmakar C., Yan C., Palaniswami M., Liu C. (2016). Classification of 5-S epileptic EEG recordings using distribution entropy and sample entropy. *Frontiers in Physiology*.

[28] Acharya U. R., Fujita H., Sudarshan V. K., Bhat S., Koh J. E. (2015). Application of entropies for automated diagnosis of epilepsy using EEG signals: a review. *Knowledge-Based Systems*.

[29] Daoud H., Bayoumi M. A. (2019). Efficient epileptic seizure prediction based on deep learning. *IEEE transactions on biomedical circuits and systems*.

[30] Hahnloser R. H. R., Sarpeshkar R., Mahowald M. A., Douglas R. J., Seung H. S. (2000). Digital selection and analogue amplification coexist in a cortex-inspired silicon circuit. *Nature*.

[31] Karnati M., Seal A., Sahu G., Yazidi A., Krejcar O. (2022). A novel multi-scale based deep convolutional neural network for detecting COVID-19 from X-rays. *Applied Soft Computing*.

[32] Alakus T. B., Turkoglu I. (2020). Emotion recognition with deep learning using GAMEEMO data set. *Electronics Letters*.

[33] Alakus T. B., Gonen M., Turkoglu I. (2020). Database for an emotion recognition system based on EEG signals and various computer games–GAMEEMO. *Biomedical Signal Processing and Control*.

[34] Dogan A., Akay M., Barua P. D. (2021). PrimePatNet87: prime pattern and tunable q-factor wavelet transform techniques for automated accurate EEG emotion recognition. *Computers in Biology and Medicine*.

[35] Pan B., Zheng W. (2021). Emotion recognition based on EEG using generative adversarial nets and convolutional neural network. *Computational and Mathematical Methods in Medicine*.

[36] Xing X., Li Z., Xu T., Shu L., Hu B., Xu X. (2019). SAE+ lstm: a New framework for emotion recognition from multi-channel EEG. *Frontiers in Neurorobotics*.

